# Atmospheric Oxidation Mechanism and Kinetic Studies for OH and NO_3_ Radical-Initiated Reaction of Methyl Methacrylate

**DOI:** 10.3390/ijms15035032

**Published:** 2014-03-20

**Authors:** Rui Gao, Ledong Zhu, Qingzhu Zhang, Wenxing Wang

**Affiliations:** 1 Environment Research Institute, Shandong University, Ji’nan 250100, China; E-Mails: sdgaorui@hotmail.com (R.G.); wxwang@sdu.edu.cn (W.W.); 2 School of Chemistry and Chemical Engineering, Shandong University, Ji’nan 250100, China; E-Mail: georgerui@sohu.com

**Keywords:** atmospheric oxidation, methyl methacrylate, rate constants, reaction mechanism

## Abstract

The mechanism for OH and NO_3_ radical-initiated oxidation reactions of methyl methacrylate (MMA) was investigated by using density functional theory (DFT) molecular orbital theory. Geometrical parameters of the reactants, intermediates, transition states, and products were fully optimized at the B3LYP/6-31G(d,p) level. Detailed oxidation pathways were presented and discussed. The rate constants were deduced by the canonical variational transition-state (CVT) theory with the small-curvature tunneling (SCT) correction and the multichannel Rice-Ramspergere-Kassele-Marcus (RRKM) theory, based on the potential energy surface profiles over the general atmospheric temperature range of 180–370 K. The calculated results were in reasonable agreement with experimental measurement.

## 1. Introduction

Methyl methacrylate (MMA, CH_3_COOCH_2_CH_3_) is widely used in manufacture of resins and plastics [1–3]. Currently, over 2.04 × 10^9^ kg of MMA has been produced by industrial processes [4]. Since the increasing use of MMA, the emission into the atmosphere may greatly rise. The high vapor pressure (3.9 × 10^3^ Pa at 293 K) indicates that MMA exists mainly in the gas phase under the general atmospheric conditions [5,6]. Neurological symptoms have been reported in humans following acute exposure to methyl methacrylate [7,8]. Fetal abnormalities have been reported in animals exposed to methyl methacrylate by injection and inhalation [9]. Once released into the atmosphere, the unsaturated MMA may be oxidized by OH radicals during daytime, nitrate radicals (NO_3_), and ozone molecules during the nighttime [10,11]. The most important oxidation degradation of MMA is initiated by reaction with OH radicals in the atmosphere. However, as the formation of OH mainly takes place in daytime, OH concentration rapidly decreases after sunset. As a consequence, reactions with OH radicals only occur during the day. NO_3_ radical undergoes rapid photolysis upon absorption of radiation. During daytime, concentration of NO_3_ radicals is very low. Therefore, daytime NO_3_ chemistry is expected to be unimportant for the MMA. However, NO_3_ has been identified and measured in the nighttime atmosphere, especially in some seriously polluted regions. The reactions with NO_3_ will dominate the loss pathway of MMA during nighttime. Moreover, in certain locations during certain times of the year, reactions with Cl atoms may also be important. These atmospheric oxidation reactions may contribute to the formation of the secondary photooxidants and aerosols in the troposphere.

The large emission, high volatility, and toxicity of MMA make it a potential important source of environmental concern in the atmosphere. The previous studies on the atmospheric reaction of MMA mainly focus on obtaining kinetic parameters. Low pressure rate constant for the reaction of MMA with OH radicals has been determined using the discharge flow-laser induced fluorescence (DF/LIF) technique at the room temperature [12]. The rate constants for the reactions of MMA with OH radicals and Cl atoms were carried out using the relative rate methods at the ambient temperature and pressure conditions [13], giving *k*_OH_ = (4.15 ± 0.32) × 10^−11^ cm^3^ molecule^−1^ s^−1^, and *k*_Cl_ = (2.82 ± 0.93) × 10^−10^ cm^3^ molecule^−1^ s^−1^. Arrhenius formula over the temperature range of 253–374 K was derived as *k*(*T*)(MMA + OH) = (2.5 ± 0.8 × 10^−12^) exp((825 ± 55)/*T*) cm^3^ molecule^−1^ s^−1^. The rate constants for the gas-phase reactions of a series of acrylate methyl (including MMA) with O_3_ were determined using smog chamber techniques [14] at 1.01 × 10^4^ Pa and 294 ± 2 K. The rate constant of MMA was obtained as *k*_O3_ = (6.7 ± 0.9) × 10^−18^ cm^3^ molecule^−1^ s^−1^. Recently, the reactions of MMA with OH radicals in the presence of NOx were studied in environmental chamber [15], revealing that the primary products are methyl pyruvate (92% ± 16%) and methanal (87% ± 12%). However, due to the scarcity of efficient detection schemes for radical intermediate species and commercially available standards, the atmospheric reaction mechanism of MMA is still unclear. In this work, quantum chemical and direct kinetic calculations were performed to elucidate the reaction mechanism of the OH and NO_3_ radical-initiated atmospheric oxidation of MMA.

## 2. Results and Discussion

To confirm the reliability of calculated results, the geometries and vibrational frequencies of CH_3_COOCH_2_CH_3_, CH_3_CHO, and CH_3_CH=CH_2_ were calculated at the B3LYP/6-31G(d,p) level. The results are in reasonable accordance with the corresponding experimental values and the discrepancy remains within 1% for geometrical parameters and 4% for vibrational frequencies [16,17].

### 2.1. Reaction Mechanism

**Figure 1.**
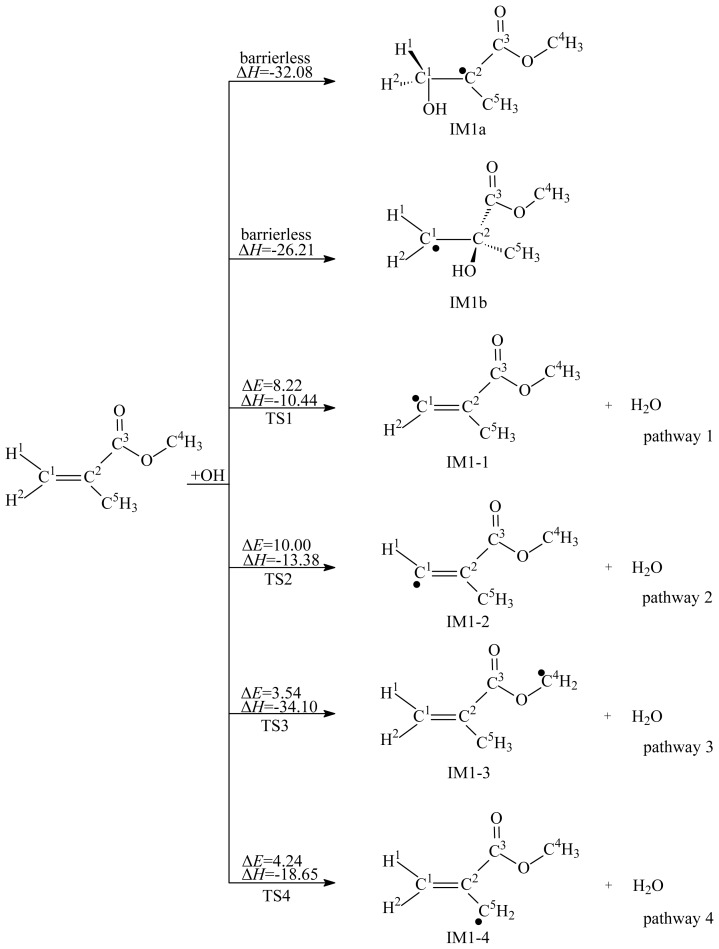
OH radical-initiated reaction schemes embedded with the potential barriers Δ*E* (in kcal/mol) and reaction heats Δ*H* (in kcal/mol, 0 K).

**Figure 2.**
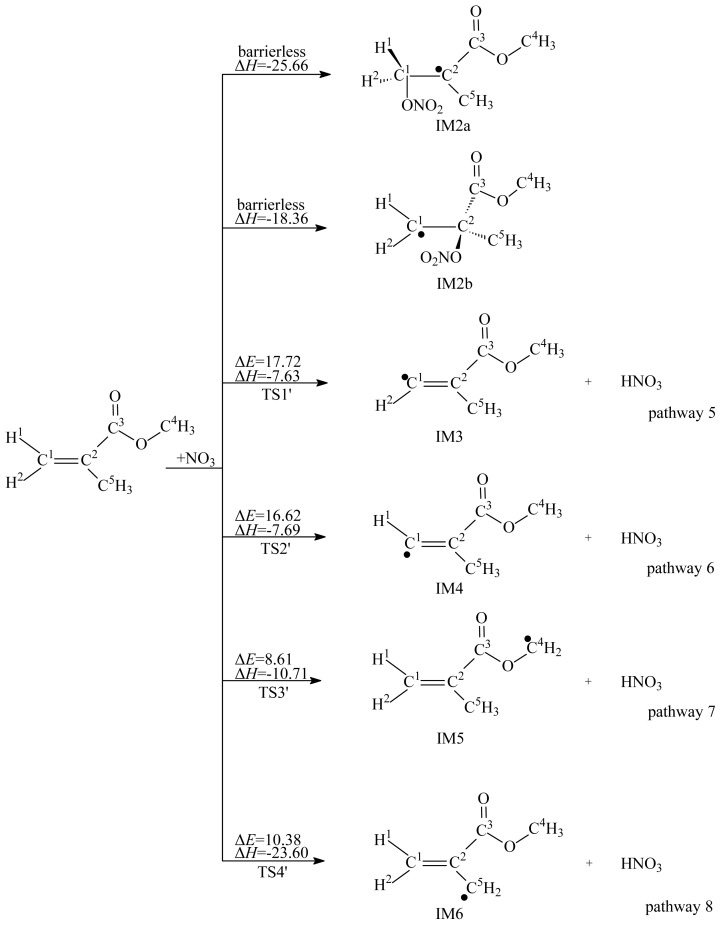
NO_3_ radical-initiated reaction schemes embedded with the potential barriers Δ*E* (in kcal/mol) and reaction heats Δ*H* (in kcal/mol, 0 K).

**Figure 3.**
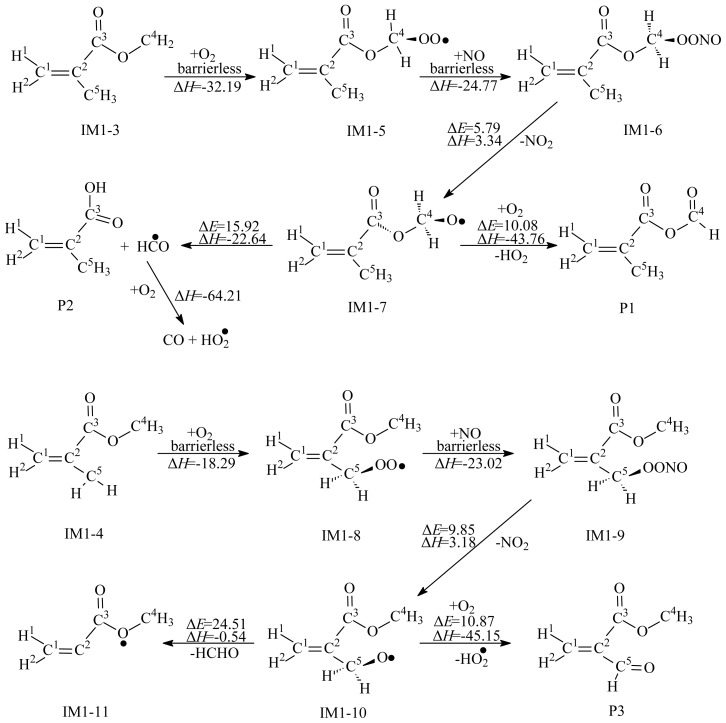
Secondary reaction of IM1–3 and IM1–4. Unit: kcal/mol. Δ*E*: the potential barriers; Δ*H*: reaction heats (0 K).

**Figure 4.**
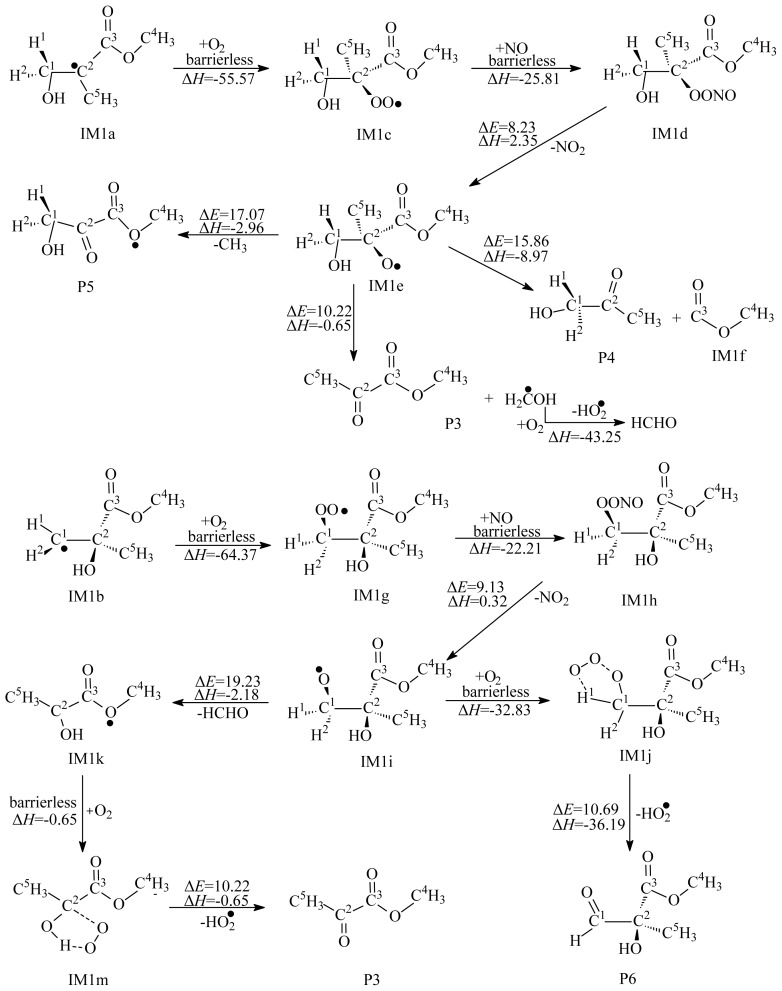
Secondary reaction of IM1a and IM1b. Unit: kcal/mol. Δ*E*: the potential barriers; Δ*H*: reaction heats (0 K).

**Figure 5.**
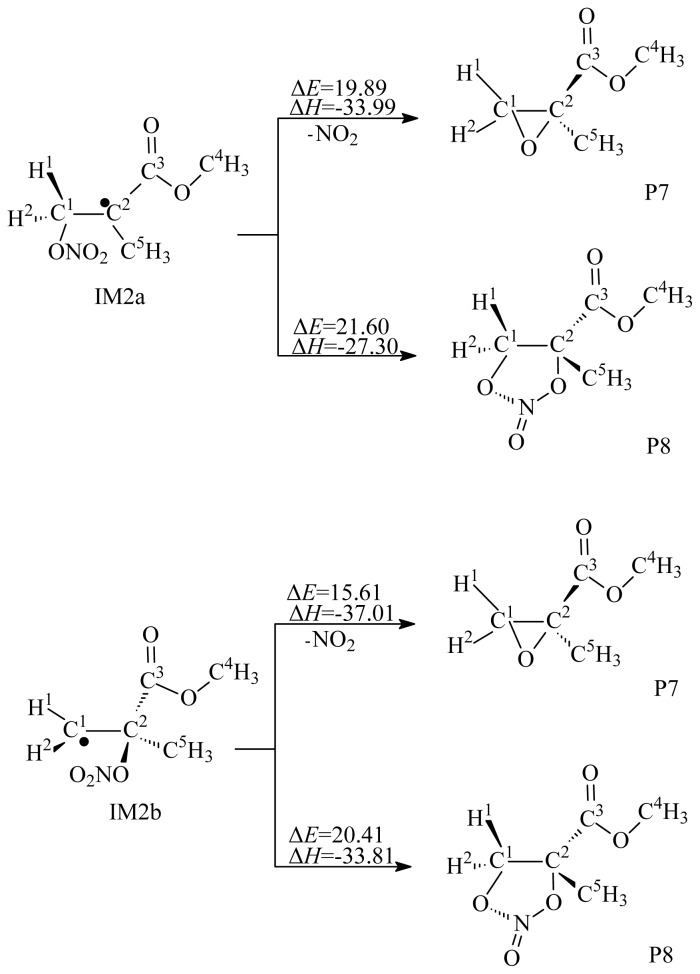
Decomposition and isomerization reactions of IM2a and IM2b. Unit: kcal/mol. Δ*E:* the potential barriers; Δ*H*: reaction heats (0 K).

**Figure 6.**
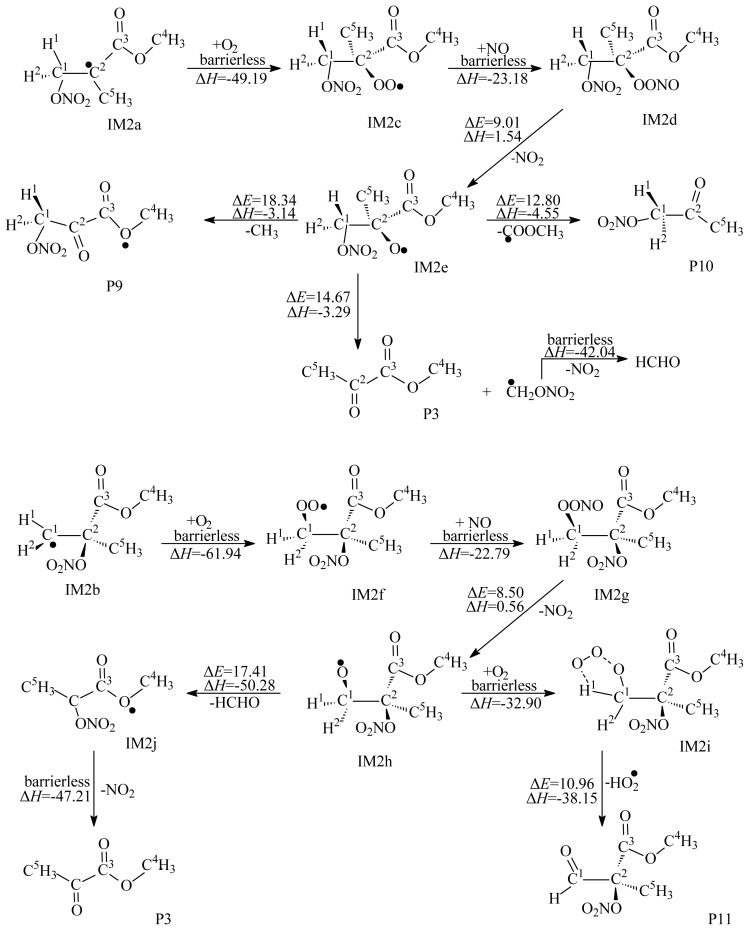
Secondary reaction of IM2a and IM2b. Unit: kcal/mol. Δ*E*: the potential barriers; Δ*H*: reaction heats (0 K).

The reactions of MMA with OH and NO_3_ radicals involve two kinds of pathways: H abstraction from MMA and addition of OH or NO_3_ radical to the C=C bond. The reaction schemes embedded with the potential barriers and reaction enthalpies are depicted in Figures 1 and 2. For convenience of description, the C and H atoms in MMA are labeled, as depicted in Figures 1–6.

#### 2.1.1. H Abstraction by OH and NO_3_ Radicals

As shown in Figures 1 and 2, there are eight H atoms in the MMA molecule. Since the C^2^–C^5^ and O–C^4^ bonds are rotatable, three H atoms bonded to C^4^ are equivalent, and three H atoms bonded C^5^ are equivalent. So, there are four kinds of H atoms in the MMA molecule, indicating that four possible pathways can be identified: H abstraction from the C^1^–H^1^, C^1^–H^2^, C^4^–H, and C^5^–H bonds. The products of H abstraction by OH or NO_3_ radicals are IM1–1, IM1–2, IM1–3, and IM1–4. Eight transition states were located (TS1, TS2, TS3, and TS4 for H abstraction by OH radicals and TS1′, TS2′, TS3′, and TS4′ for H abstraction by NO_3_ radicals), and each one was identified with only one imaginary frequency. The reaction schemes embedded with the potential barriers and reaction heats are depicted in Figures 1 and 2. All the H abstraction processes are exothermic. The potential barriers of pathways 3 and 4 are lower than that of pathways 1 and 2. In addition, pathways 3 and 4 are more exothermic than pathways 1 and 2. Thus, the thermodynamically favored H abstraction pathways are pathways 3 and 4. The resulting IM1–3 and IM1–4 are the main H abstraction products. Similarly, pathways 7 and 8 are thermodynamically favored H abstraction pathways for the reaction of MMA with NO_3_ radicals. Two same products, IM1–3 and IM1–4, can be yielded from pathways 7 and 8. Therefore, IM1–3 and IM1–4 are important intermediates produced in the oxidation process of MMA initiated by OH and NO_3_ radicals.

IM1–3 and IM1–4 are activated radicals and can further react with the ubiquitous oxygen molecules in the atmosphere to form two organic peroxy radicals, IM1–5 and IM1–8. To evaluate the nature of the entrance channel for the formation of the organic peroxy radicals of IM1–5 and IM1–8, we examined the potential along the reaction coordinate, especially to determine whether there is a well-defined transition state or if the process proceeds via a loose transition state without a barrier, depicted in Figure 3. The profiles of the potential energy surface were scanned by varying the newly formed C^4^–O or C^5^–O bond. We found no energy exceeding the C^4^–O or C^5^–O bond dissociation threshold along the reaction coordinate. This shows that the reactions of IM1–3 and IM1–4 with O_2_ proceed via a barrierless association. The processes are strongly exothermic by 32.19 and 18.29 kcal/mol.

In the troposphere, IM1–5 or IM1–8 will react immediately with ubiquitous NO. The entrance channel of the reactions is exoergic, leading to two vibrationally excited intermediates (denoted as IM1–6 and IM1–9), which promptly react via unimolecular decomposition. Unimolecular decompositions of IM1–6 and IM1–9 occur via cleavage of the O-O bond, forming NO_2_ and two alkoxy radicals IM1–7 and IM1–10. The two unimolecular decomposition processes are endothermic with high potential barrier. IM1–7 can further react with O_2_, followed by loss of HO_2_, to form CH_2_=C(CHO)COOCH_3_ (P1) and HO_2_, or via unimolecular decomposition and H migration to form CH_2_=C(CH_3_)C(O)OC(O)OH (P2). The decomposition of IM1–10 has a high barrier of 24.51 kcal/mol and cannot occur under the general atmospheric conditions. The reaction with O_2_ is the primary removal pathway for IM1–10, and the products are CH_2_=C(CHO)COOCH_3_ (P3) and HO_2_.

#### 2.1.2. Addition of OH to MMA

Two carbon atoms in the C=C bond of MMA are not equivalent, thus, OH radicals can attack C^1^ or C^2^ atom to form two different adducts, IM1a and IM1b, as shown in Figure 1. The OH-MMA adducts, IM1a and IM1b, could easily further react with O_2_/NO in the atmosphere and form intermediates IM1d and IM1h, respectively. As shown in Figure 4, the two addition steps are barrierless and strongly exothermic. Then, the energy-rich intermediates IM1d and IM1h can decompose to yield IM1e and IM1i. The two decomposition processes are endothermic with high potential barriers. Three possible unimolecular decomposition pathways from IM1e were identified. The first pathway occurs via H migration and breaking of C^2^–C^5^ bond to yield P3. The barrier height is 10.22 kcal/mol. The second and third decomposition pathways are cleavage of C^1^–C^2^ and C^2^–C^3^ bonds, with barriers of 17.07 and 15.86 kcal/mol. The results show that the first decomposition pathway is energetically more favorable. Unimolecular decomposition of IM1i only occurs via cleavage of C^1^–C^2^ bond to yield IM1k. In addition, IM1i can further react with O_2_, followed by the loss of HO_2_, to yield CH_3_OC(O)C(CH_3_)(OH)CHO (P6). The decomposition of IM1i needs to overcome a barrier of 19.23 kcal/mol, therefore, the reaction with O_2_ is relatively more favorable under the general atmospheric conditions. The calculated results suggest that the main products from secondary reactions of OH-MMA adducts are P3, HO_2_ and methanal.

#### 2.1.3. Addition of NO_3_ to MMA

Similar to OH addition to MMA, the addition of NO_3_ to MMA can form two different NO_3_-MMA adducts, IM2a and IM2b, as shown in Figure 2. The scanned profile of the potential energy surface shows that the two addition reactions are barrierless. The NO_3_-MMA adducts can further react via decomposition or isomerization, as depicted in Figure 5, or be removed via reactions with O_2_/NO, as depicted in Figure 6. As shown in Figure 5, in the decomposition processes, a three-membered ring product, P7, was generated from the intermediates IM2a and IM2b via loss of NO_2_, with potential barriers of 19.89 and 15.61 kcal/mol; and a five-membered ring product, P8, was formed from the isomerization of IM2a and IM2b, with potential barriers of 21.60 and 20.41 kcal/mol. The results suggest that the decomposition and isomerization processes are competitive.

In the oxygen-rich atmosphere, the NO_3_-MMA adducts could further react with O_2_ to yield organic peroxy radicals. The detailed subsequent reactions are presented in Figure 6. The reactions of IM2a and IM2b with O_2_ are barrierless and strongly exothermic by 49.19 and 61.94 kcal/mol to form IM2c and IM2f. In the troposphere, the two intermediates, IM2c and IM2f, will further react with ubiquitous NO immediately and yield two vibrationally excited intermediates, IM2d and IM2g, respectively. Unimolecular decompositions of IM2d and IM2g result in the formation of IM2e and IM2h via loss of NO_2_. The alkoxy radical IM2e can further react via three possible unimolecular decomposition pathways, as depicted in Figure 6. Comparison of potential barriers of the three pathways shows that cleavage of the C^1^–C^2^ and C^2^–C^3^ bonds are energetically favorable, leading to the products of P3, P11 and methanal. IM2j produced from the decomposition of IM2h can further decompose to yield P3 via the loss of NO_2_. This process is exothermic by 47.21 kcal/mol. IM2j can also further react with O_2_, followed by the loss of HO_2_ to produce P11. Calculations show that the reaction of IM2j with O_2_ is energetically more favorable than the decomposition of IM2j. Comparison of the three kinds of the reaction pathways for NO_3_-MMA adducts suggests that the reaction of the NO_3_-MMA adduct with O_2_/NO is thermodynamically and energetically more favorable than their unimolecular decomposition or isomerization.

### 2.2. Rate Constant Calculations

On the basis of the profile of the potential energy surface calculated by the CCSD(T)/6-31G(d) + CF//B3LYP/6-31G(d,p) method, the individual and overall rate constants for the OH and NO_3_ radical-initiated reactions of MMA were calculated over the temperature range from 180 to 370 K, which is the typical temperature range of the troposphere. The rate constants of H abstraction from MMA were calculated by using the CVT/SCT method. The individual rate constants for the H abstraction pathways 1–8 are noted as *k*_abs_^1^–*k*_abs_^8^, respectively. For the addition pathways, the rate constants were calculated by using the multichannel RRKM method. The rate constants for addition of OH to the C^1^ and C^2^ atoms of MMA are noted as *k*_OH_^1^ and *k*_OH_^2^, respectively; and the rate constants for addition of NO_3_ to the C^1^ and C^2^ atoms are noted as *k*_NO3_^1^ and *k*_NO3_^2^, respectively. The overall rate constant for the reaction of MMA with OH radical is noted as *k*_OH_, *k*_OH_ = *k*_OH_^1^ + *k*_OH_^2^ + *k*_abs_^1^ + *k*_abs_^2^ + *k*_abs_^3^ + *k*_abs_^4^; and the overall rate constant for the reaction with NO_3_ radical is noted as *k*_NO3_, *k*_NO3_ = *k*_NO3_^1^ + *k*_NO3_^2^ + *k*_abs_^5^ + *k*_abs_^6^ + *k*_abs_^7^ + *k*_abs_^8^. Comparison of the rate constants of the H abstraction pathways and addition pathways shows that the H abstraction pathways can be negligible for their little contribution under the general atmospheric conditions.

At 298 K, the calculated overall rate constants for the reactions of MMA with OH and NO_3_ radicals are 4.36 × 10^−11^ and 3.64 × 10^−15^ cm^3^ molecule^−1^ s^−1^, excellently agreed with the available experimental values [13,18,19]. The good agreement infers that the calculated other rate constants are reasonable. The calculated overall rate constants are fitted over the temperature range of 180–370 K, and Arrhenius formulas are given in units of cm^3^ molecule^−1^ s^−1^:

(1) $$k(T)(\text{MMA}+\text{OH})=(1.83\times 10^{-12})\text{exp}(945.58/T)$$

(2) $$k(T)(\text{MMA}+{\text{NO}}_{3})=(6.75\times 10^{-16})\text{exp}(502.48/T)$$

To assess the impact to the environment, it is critical to known the atmospheric lifetime of MMA. The global average concentration OH radical (*c*_OH_) in daytime is 2 × 10^6^ molecule/cm^3^ [20], and the typical concentration of NO_3_ radicals (*c*_NO3_) in the continental boundary layer is 5 × 10^8^ molecule/cm^3^ [21]. According to the rate constants of the reactions of MMA with OH and NO_3_ radicals, using the expression:

(3) $$\tau_{\text{OH}}=\frac{1}{k_{\left(\text{OH}+\text{MMA}\right)}\times c_{\text{OH}}}$$

(4) $$\tau_{{NO}_{3}}=\frac{1}{k_{\left({\text{NO}}_{3}+\text{MMA}\right)}\times c_{{\text{NO}}_{3}}}$$

The atmospheric lifetimes of MMA determined by OH and NO_3_ radicals are 3 h and 6.5 days, respectively. The obtained lifetimes here can be comparable to 2–10 h for OH radicals [21] and 6 days for NO_3_ radicals in the previous work [14]. Therefore, MMA is likely to be removed quickly by the reaction with OH radicals near to their emission sources. However, since the atmospheric lifetime of MMA were determined both by the rate constant of the reactions with radicals and the concentration of radicals, in some seriously polluted regions, the reaction with NO_3_ radicals also may contribute to the removal of MMA in the atmosphere. Moreover, in some coastal areas where the concentration of Cl atoms can reach a peak value of 1 × 10^5^ molecule/cm^3^ [22,23], the MMA may be quikly removed by the reaction with Cl atoms [15].

## 3. Computational Methods

All the calculations were performed with the Gaussian 03 software package (Gaussian, Inc., Wallingford, CT, USA). Geometrical parameters of the reactants, intermediates, transition states, and products were fully optimized at the B3LYP level with a standard basis set 6-31G(d,p). The nature of stationary points, the zero-point energy (ZPE), and the thermal contribution to the free energy of activation were determined by the vibrational frequency calculation at the same level. For each transition state, the intrinsic reaction coordinate (IRC) calculation was performed using the same electronic structure theory to confirm it was connected to the right minima along the reaction pathway. The DFT geometries were then used in the single-point energy calculations at the frozen-core second-order Møller-Plesset perturbation theory (MP2) and the coupled-cluster theory with single and double excitations including perturbative corrections for the triple excitations (CCSD(T)) with several basis sets. Each single-point energy was further corrected using a factor, CF. This factor was determined from the energy difference between the MP2/6-311++G(d,p) and MP2/6-31G(d) levels, and the ranges of CF value are 0.001592 to 0.3571924 Hartree in the reactions of MMA with OH radical, and 0.010456 to 0.2470771 Hartree in the reactions of MMA with NO_3_ radical. The values of calculated energies at the CCSD(T)/6-31G(d) level were then corrected by the CFs, corresponding to the CCSD(T)/6-31G(d) + CF level of theory [24,25]. Potential barriers (Δ*E*, Δ*E* = *E*_transition state_ − ∑*E*_reactants_) and reaction heats (Δ*H*, Δ*H* = ∑*E*_products_ − ∑ _reactants_) were determined in each channel.

Depending on different reaction types, two methods were used to calculate the rate constants. For the abstraction pathways, the widely used canonical variational transition-state theory with the small curvature tunneling (CVT/SCT) correction was adopted. The theoretical rate constants and their temperature dependence were calculated by using the Polyrate 9.3 program (University of Minnesota, Minneapolis, MN, USA). Actually, the CVT/SCT method has been successfully used in dealing with several bimolecular reactions [26]. For the addition pathways, the rate constants were calculated using multichannel Rice-Ramspergere-Kassele-Marcus (RRKM) theory. This method has been successfully used in the previous works for several addition reactions [27].

## 4. Conclusions

A theoretical study was performed on the mechanism of OH and NO_3_ radical-initiated reactions of methyl methacrylate. The rate constants were calculated by using the CVT/SCT and multichannel RRKM method. Several specific conclusions can be drawn from this study:

(1) The mechanism of OH and NO_3_ radical-initiated reactions of MMA includes H abstraction pathways and the addition pathways, and the H abstraction pathways can be negligible because of their little contribution under the general atmospheric conditions.
(2) The OH-MMA and NO_3_-MMA adducts are open-shell activated radical intermediates, and can further react with O_2_/NO in the atmosphere. For the OH radical-initiated reaction, the main products are CH_3_C(O)C(O)OCH_3_, HO_2_ and methanal, consistent with the experimental results [15]. For the NO_3_ radical-initiated oxidation, the reaction of the NO_3_-MMA adduct with O_2_/NO is thermodynamically and energetically more favorable than their unimolecular decomposition or isomerization under the general atmospheric conditions and the main products are CH_3_C(O)C(O)OCH_3_, CH_3_C(O)CH_2_NO_3_ and methanal.

The calculated overall rate constants match well the available experimental values. The atmospheric life times of MMA determined by OH radicals and NO_3_ radicals are 3 h and 6.5 days. NO_3_-initiated oxidation reaction contributes little to the atmospheric losses of MMA except in polluted regions.
